# Microbial degradation mechanisms of the neonicotinoids acetamiprid and flonicamid and the associated toxicity assessments

**DOI:** 10.3389/fmicb.2024.1500401

**Published:** 2024-11-05

**Authors:** Shilei Sun, Jingjing Guo, Zhi Zhu, Jiangsheng Zhou

**Affiliations:** ^1^The Key Laboratory of Biotechnology for Medicinal Plants of Jiangsu Province and School of Life Science, Jiangsu Normal University, Xuzhou, China; ^2^School of Life Science and Environmental Engineering, Nanjing Normal University Zhongbei College, Zhenjiang, China

**Keywords:** neonicotinoid insecticide, acetamiprid, flonicamid, biodegradation, molecular mechanism

## Abstract

Extensive use of the neonicotinoid insecticides acetamiprid (ACE) and flonicamid (FLO) in agriculture poses severe environmental and ecological risks. Microbial remediation is considered a feasible approach to address these issues. Many ACE-and FLO-degrading microorganisms have been isolated and characterized, but few reviews have concentrated on the underlying degradation mechanisms. In this review, we describe the microbial degradation pathways of ACE and FLO and assess the toxicity of ACE, FLO and their metabolites. Especially, we focus on the enzymes involved in degradation of ACE and FLO, including cytochrome P450s, nitrile hydratases, amidases, and nitrilases. Those studies reviewed here further our understanding of the enzymatic mechanisms of microbial degradation of ACE and FLO, and aid in the application of microbes to remediate environmental ACE and FLO contamination.

## 1 Introduction

Neonicotinoid insecticides (NEOs) emerged in the 1990s as fourth-generation pesticides following organophosphates, pyrethroids, and carbamates (Hladik et al., 2018). Because of their high efficacy against insect pests (aphids, whiteflies, beetles and other soil pests) and low acute toxicity toward mammals, NEOs have become the most popular insecticides in the world, registered in 120 countries and accounting for 25% of the global pesticide market (Tu et al., 2023; Jeschke et al., 2011). They are widely used in plantation areas of rice, wheat, maize, soybean, cotton, sugar beet, apple, and potato (Randhawa, 2024; Li et al., 2020). The application methods of NEOs are versatile, including foliar sprays, soil drenches and seed treatment (Goulson, 2013). However, after killing the pests, NEOs persist in crops, giving rise to food safety concerns. Two cross-sectional studies (the U.S. Congressional Cafeteria Study and Hangzhou China Study) provide evidence that neonicotinoids have become ubiquitous in the global food supply (Thompson et al., 2020). Moreover, only a small portion (on average 5%) of the NEO is absorbed by the crop and the remainder passes into soil and water, resulting in a chain of ecological and environmental issues (Wood and Goulson, 2017). For example, NEOs have diverse degradation half-lives in soil, ranging from tens to hundreds of days; their presence has a negative impact on soil invertebrates (Ge et al., 2018). A review of 214 acute and chronic toxicity tests of NEOs toward aquatic insects indicated that Ephemeroptera (mayflies), Trichoptera (caddisflies), and Diptera (flies including chironomid midges) were the most sensitive taxa to NEOs (Morrissey et al., 2015). Moreover, NEOs are the subject of growing concern over their adverse health effects on humans, including cancer, chronic disease, birth defects, and infertility (Zhang et al., 2018; Zhang et al., 2021). Thus, NEO pollution is an increasing global challenge.

Abundant remediation strategies, including physiochemical, microbial, and phytoremediation, have been developed to resolve NEO pollution. Microbial remediation is widely accepted by means of the advantages of economic efficiency and environmental friendliness. Many NEO degradation microorganisms have been isolated, belonging to the genera *Rhodococcus*, *Stenotrophomonas*, *Variovorax*, *Microvirga*, *Pseudomonas*, *Bacillus*, and *Ochrobactrum* and their degradation mechanisms are characterized (Anjos et al., 2021; Gautam and Dubey, 2023; Ahmad et al., 2021). The ACE, belonging to the first-generation NEOs and FLO, belonging to the latest-generation NEOs all contain a similar pharmacophore, which is often the initial site of microbial degradation. Investigations concerned with the mechanisms of ACE and FLO degradation are relatively adequate and have increased in recent years. Therefore, in this review, microbial degradation mechanisms of NEOs ACE and FLO are summarized, including the degradation pathways and the degradation enzymes, and toxicity assessments are discussed. This article reviewed here deepens our understanding of the enzymatic mechanisms of microbial degradation of ACE and FLO, and furthers the application of microbes to remediate environmental ACE and FLO contamination.

## 2 Microbial degradation pathways of ACE and FLO

### 2.1 ACE degradation

**Figure 1:**
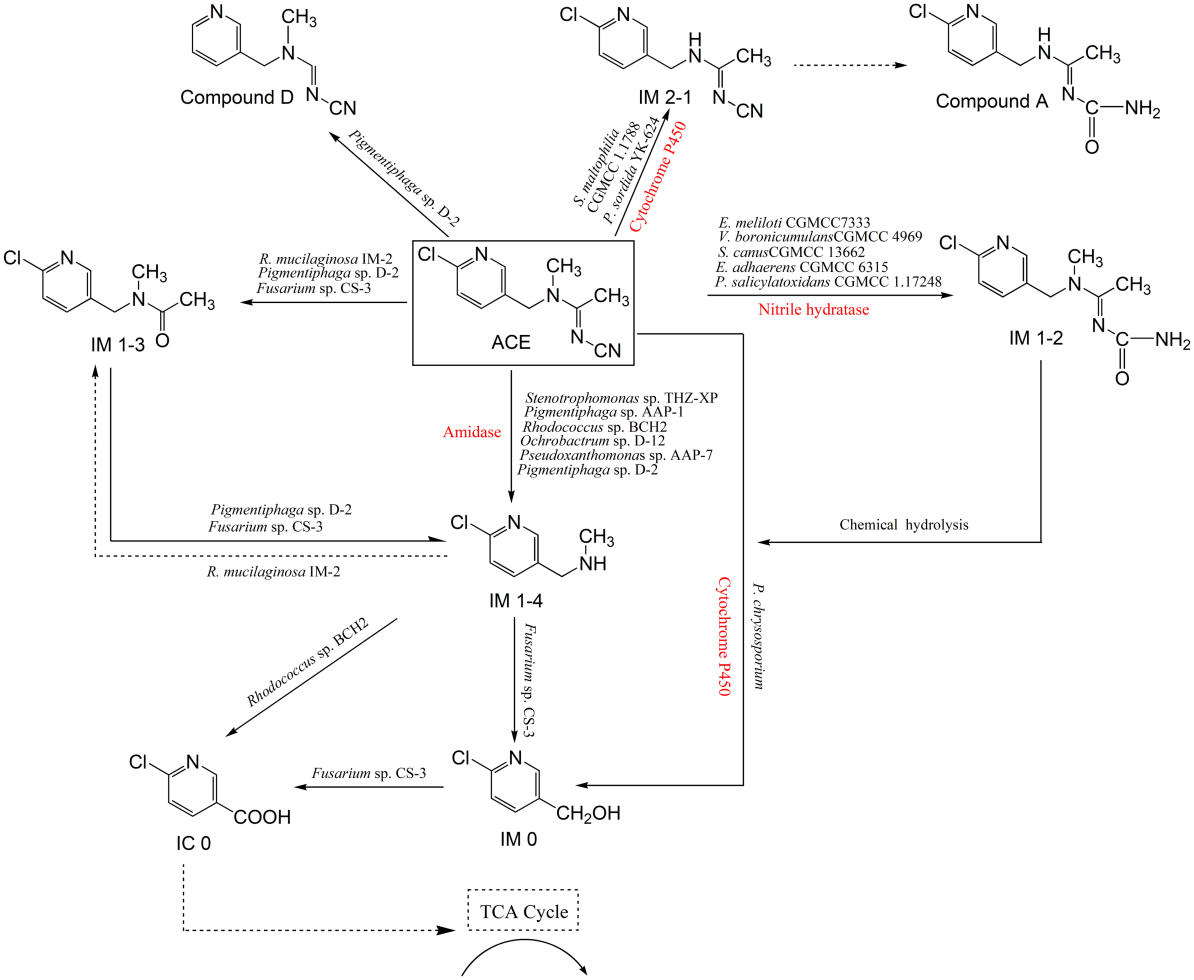
Microbial degradation pathways of ACE and associated enzymes.

There are multiple microbial degradation pathways of ACE, which are mainly confirmed by the identification of relevant metabolites (Figure 1). The metabolite *N*-[(6-chloropyridin-3-yl) methyl]-*N*-methylacetamide (also called IM 1–3), formed by oxidative cleavage of the cyanamine group of ACE, was first reported in the yeast *Rhodotorula mucilaginosa* IM-2 (Dai et al., 2010). IM 1–4 has been identified as the major metabolite of ACE in mice, honeybees and spinach. In bacteria such as *Stenotrophomonas* sp. THZ-XP, *Pigmentiphaga* sp. AAP-1, *Ochrobactrum* sp. D-12, and *Pseudoxanthomonas* sp. AAP-7, ACE can be degraded directly to IM 1–4, with no emergence of IM 1–3 (Tang et al., 2012; Wang et al., 2013a; Wang et al., 2013b; Wang et al., 2013c.) *Pigmentiphaga* sp. AAP-1 can use ACE as its sole carbon, nitrogen and energy source for growth, and it metabolized 100 mg/L ACE within 2.5 h, which exhibited the highest ACE degradation ability (Wang et al., 2013b). Moreover, a dechlorinated and demethylated product, compound D, was partially confirmed by detecting chlorine ion release in ACE degradation in *Pigmentiphaga* sp. D-2 (Yang et al., 2013).

The fungus *Fusarium* sp. CS-3 can further degrade IM 1–4 to 6-chloro-3-pyridinemethanol (IM 0), which has been detected in rats and plants as an ACE metabolite (Shi et al., 2018). In contrast, *Phanerochaete chrysosporium* can directly degrade ACE to IM 0 (Wang et al., 2019a). Subsequently, the generated IM 0 was further oxidized to 6-chloronicotinic acid (IC 0) by *Fusarium* sp. CS-3 (Shi et al., 2018). The bacterium *Rhodococcus* sp. BCH2 can directly degrade IM 1–4 to IC 0 by oxidative cleavage of the methylamino group with no formation of the intermediate IM 0 (Phugare and Jadhav, 2015). IC 0 is a common intermediate product in neonicotinoids metabolism, occurred in that bees metabolize ACE and IMI degradation process by *Mycobacterium* sp. strain MK6, which can be continuously degraded by *Fusarium* sp. CS-3 to undetectable products (Shi et al., 2018; Kandil et al., 2015).

The bacterium *Stenotrophomonas maltophilia* CGMCC 1.1788 and the fungus *Phanerochaete sordida* YK-624 degrade ACE by demethylation to give the metabolite (*E*)-*N*^1^-[(6-chloro-3-pyridyl)-methyl]-*N*^2^-cyano-acetami-dine (IM 2–1), which is also reported in the metabolism of ACE in spinach and honeybees (Chen et al., 2008; Wang et al., 2012). In spinach, IM 2–1 was further metabolized to compound A, however, this pathway has not been discovered in microbes (Yang et al., 2013). ACE has a cyano pharmacophore, which plays a crucial role in its insecticidal activity (Casida, 2011). *Ensifer meliloti* CGMCC 7333 degrades ACE beginning with this moiety, and hydrates the *N*-cyanoimine group to the *N*-carbamoylimine metabolite (IM 1–2) (Zhou et al., 2014). Var*iovorax boronicumulans* CGMCC 4969, *Streptomyces canus* CGMCC 13662, *Ensifer adhaerens* CGMCC 6315, and *Pseudaminobacter salicylatoxidans* CGMCC 1.17248 were subsequently isolated and reported to degrade ACE via an identical pathway (Sun et al., 2017; Guo et al., 2019; Sun et al., 2019; Guo et al., 2021). IM 1–2 is not stable, and it spontaneously degrades to the major metabolite IM 1–4 via hydrolysis of the *N*-carbamoylimine group to give the derivatives ACE-NH and ACE-NH_2_, which degradation process is not involved in microorganisms (Zhou et al., 2014).

### 2.2 FLO degradation

**Figure 2:**
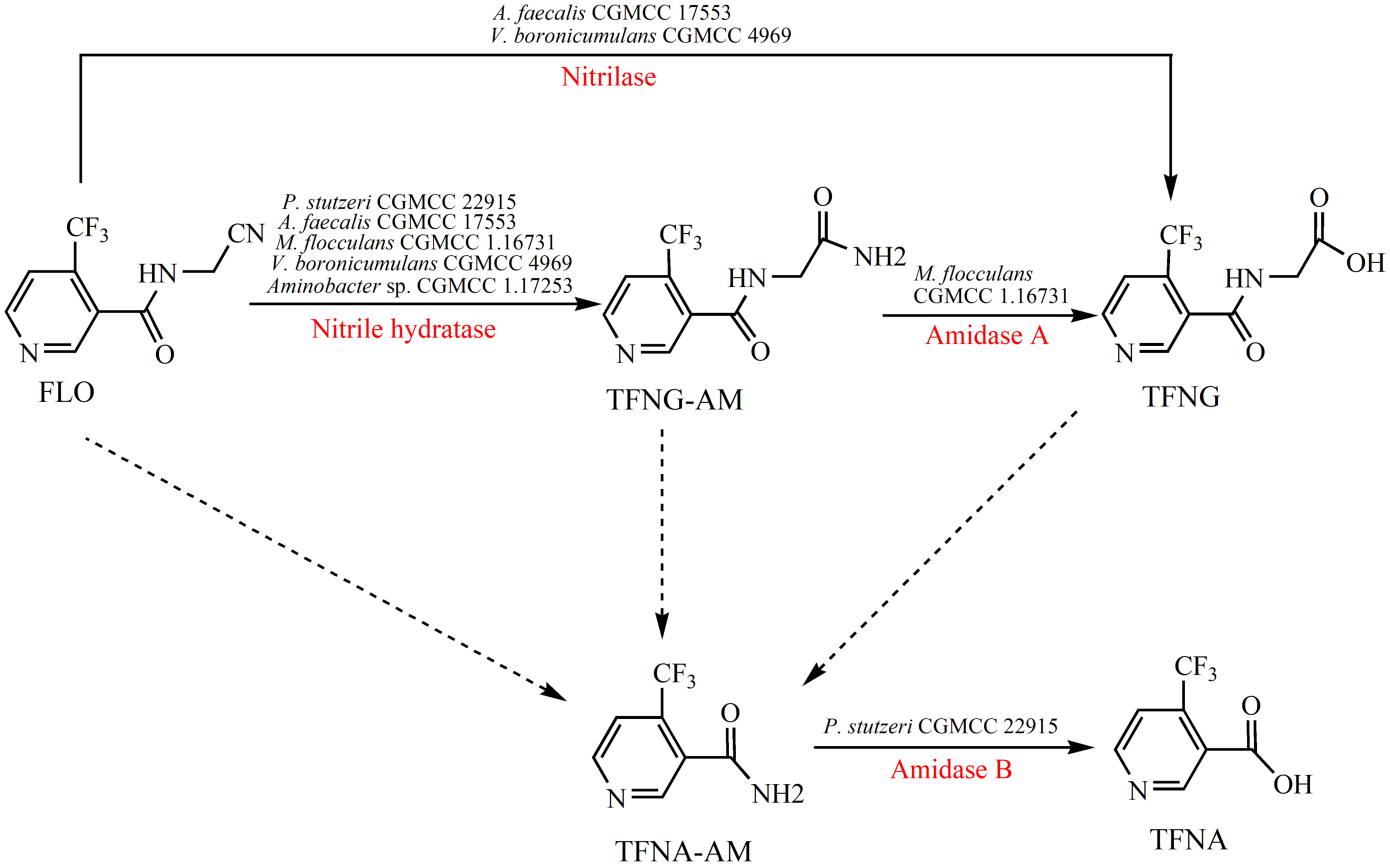
Microbial degradation pathways of FLO and associated enzymes.

The metabolism of FLO in plants has been investigated adequately, but there is limited information about microbial degradation of FLO (Figure 2). *Alcaligenes faecalis* CGMCC 17553 degraded 98.8% of 209.7 mg/L FLO in 96 h with the formation of two metabolites – 4-(trifluoromethyl) nicotinol glycine (TFNG) and *N*-(4-trifluoromethylnicotinoyl) glycinamide (TFNG-AM) – by hydrolyzing the cyano moiety of FLO (Yang et al., 2019). Those metabolites were also detected in FLO degradation in *Microvirga flocculans* CGMCC 1.16731 and Var*iovorax boronicumulans* CGMCC 4969 (Zhao et al., 2020; Jiang et al., 2022). Although those strains all degraded FLO to the same metabolites, but different degradation mechanisms occurred. *A. faecalis* CGMCC 17553 degraded FLO to TFNG and TFNG-AM via a nitrilase pathway, whereas *M. flocculans* CGMCC 1.16731 via a NHase/amidase pathway. In *V. boronicumulans* CGMCC 4969, both NHase/amidase and nitrilase pathway were discovered (Zhao et al., 2020; Jiang et al., 2022). However, in *Ensifer meliloti* CGMCC 7333, *E. adhaerens* CGMCC 6315, and *Aminobacter* sp. CGMCC 1.17253, the sole degradation product was TFNG-AM, indicating the those strains degraded FLO via the NHase pathway (Yang et al., 2021a; Yang et al., 2021b; Zhao et al., 2021b). Resting cells of *E. adhaerens* CGMCC 6315 exhibited splendid degradation potential, eliminating 92% of 199.4 mg/L FLO within 24 h, and both free and immobilized (by gel beads, using calcium alginate as a carrier) cells effectively degraded FLO in surface water (Zhao et al., 2021b). 4-Trifluoromethylnicotinamide (TFNA-AM) is the main intermediate in the metabolism of FLO in crops, produced by oxidative cleavage of the carbon–nitrogen single bond of FLO, TFNG-AM and TFNG that adjoins their pharmacophore; no pathway for these reactions has been identified in microbes. The bacterium *Pseudomonas stutzeri* CGMCC 22915 degraded TFNA-AM to 5-trifluoromethylnicotinic acid (TFNA) by hydrolysis of the amide group of TFNA-AM, with a degradation rate of 60.0%; this is the only report of microbial degradation of TFNA-AM (Jiang H. Y. et al., 2023).

## 3 Degradation enzymes for ACE and FLO

### 3.1 Cytochrome P450s

The cytochrome P450s (CYPs) are a heme–mercaptide protein superfamily, which is concerned with the oxidation and degradation of many exogenous compounds (He et al., 2024) and plenty of evidence shows that CYPs participate in the degradation of NEOs. In mammal, CYP3A4 isolated from human liver was reported to oxidize IMI, THX, and CLO (Shi et al., 2009). In insects, the development of enhanced resistance to insecticides is related to CYPs. Pym et al. (2023) reported that a single point mutation in, and overexpression of, *Bemisia tabaci* CYP6CM1 resulted in improved resistance to NEOs. Considering ACE degradation, *S. maltophilia* CGMCC 1.1788 and *P. sordida* YK-624 were reported to demethylate ACE to generate IM 2–1; addition of the CYP inhibitor PBO and piperonyl butoxide apparently decreased ACE degradation rate, which indicated that CYP plays an important role in the N-demethylation of ACE (Chen et al., 2008; Wang et al., 2012). Wang et al. (2022) evaluated the differentially expressed genes of *P. sordida* YK-624 under ACE-degrading conditions by RNA sequencing. They discovered 11 differentially expressed genes characterized as cytochrome P450s were upregulated, and these genes were determined to be particularly important for ACE degradation by *P. sordida* YK-624 under ligninolytic conditions.

*Phanerochaete chrysosporium* directly degrades ACE to IM 0, and this metabolic process also involves CYP. Wang et al. (2019a) used a *Saccharomyces cerevisiae* heterologous expression system to express 120 CYPs from *P. chrysosporium* ME-446. The results showed that CYP5147A3 can degrade ACE to two metabolites, *N′*-cyano-*N*-methyl acetamidine and IM 0. Mori et al. (2021) screened another isozyme, CYP5037B3, that can also degrade ACE to IM 0. Both CYPs (CYP5037B3 and CYP5147A3) can catalyze cleavage of the NEOs ACE, IMI, and THI, which have in common a chloropyridinyl moiety, by *N*-dealkylation, resulting in the formation of IM 0 and respective side-chain fragments. In addition, CYPs were discovered to play a key role in the degradation of other NEOs. Wang et al. (2019b) reported that *Phanerochaete sordida* YK-624 degraded 31% of DIN and 100% of NIT in ligninolytic conditions; addition of the CYP inhibitor 1-aminobenzotriazole markedly inhibited the degradation activity. Similar was observed for CLO degradation by *P. sordida* YK-624 (Chen et al., 2021). Confirming the CYPs that catalyze NIT, DIN, ACE, and CLO degradation in *P. sordida* is significant future work.

### 3.2 Nitrile hydratases

Nitrile hydratases (NHases; EC 4.2.1.84) are metalloenzymes that hydrolyze nitriles to the corresponding amides (Cheng et al., 2020). NHases are generally heteromeric proteins, containing *α*-and *β*-subunits. An activator protein encoded in the NHase gene cluster usually plays a vital role in the maturation of NHase by incorporating metal ions (Sun et al., 2021). However, as an increasing number of NHases are discovered, variation is apparent in the gene clusters that encode them. The NHase gene clusters from the ACE-and FLO-degrading bacteria *Ensifer meliloti* CGMCC 7333, *E. adhaerens* CGMCC 6315, Var*iovorax boronicumulans* CGMCC 4969, *Aminobacter* sp. CGMCC 1.17253, and *Pseudaminobacter salicylatoxidans* CGMCC 1.17248 are encoded in the order <*α*-subunit> < β-subunit> <activator> (Yang et al., 2021a; Zhou et al., 2014; Sun et al., 2017; Sun et al., 2019; Guo et al., 2021). Recently, Guo et al. (2024) reported an archaeal NHase derived from the halophilic archaeon A07HB70, possessing a notable feature of fused α-subunit with the activator, which exhibits significantly higher substrate and product tolerance compared with NHases derived from other sources.

The NHase from the bacterium *Streptomyces canus* CGMCC 13662 can degrade ACE to IM 1–2 and was reported to have an unusual three-subunit composition, with one α-subunit and two β-subunits; no activator protein was discovered (Guo et al., 2019). This three-subunit NHase organization was also found for FLO-degrading *Microvirga flocculans* CGMCC 1.16731 plasmid-encoded NHase, in which the two β-subunits encoding genes were separated by the α-subunit encoding gene (Zhao et al., 2020). All the reported ACE and FLO-degrading NHases are Co-type NHase. The widely accepted maturation mechanism of Co-type NHase is a “self-subunit swapping” hypothesis, first proposed for *Rhodococcus rhodochrous* J1 L-NHase (Zhou et al., 2008). However, the maturation mechanism might be different for the NHases that lack activator proteins. Guo et al. (2020) reported the maturation of *Streptomyces canus* CGMCC 13662 NHase, and a trimer (β_2_α) that was responsible for carrying and transferring Co was discovered. The Co ion was first incorporated into the α-subunit of Apo-β_2_α in a reducing environment, and, subsequently, the Co-containing-α-subunit in holo-β_2_α was exchanged with apo-Anhβ1_2_β2_2_α_2_ by a self-subunit swapping mode.

The expression of NHase may be constitutive or inducible, and for the latter, it is controlled by different regulatory factors. Amide/urea induction of NHase expression is most frequent and was first elucidated for *R. rhodochrous* J1 NHase (Komeda et al., 1996). Metal ion-induction of expression was reported for *R. rhodochrous* M8 Co-type NHase, dependent on the downstream regulator CblA, a co-responsive repressor (Lavrov et al., 2018). Carbon and nitrogen catabolite inhibition was also discovered for *R. rhodochrous* M8 NHase (Leonova et al., 2000), but the relevant molecular mechanism is unclear. Sun et al. (2019) reported low-nutrient-induced NHase expression in *Ensifer adhaerens* CGMCC 6315. A further investigation by Jiang N. D. et al. (2023) found that NtrC, a global transcriptional regulatory factor that regulates nitrogen metabolism in bacteria, induces NHase expression in ammonium-limited conditions and inhibits the expression in the presence of ammonium. This mechanism might partly account for nitrogen-mediated catabolite inhibition of NHase expression.

### 3.3 Amidases

Amidases (EC 3.5.1.4) catalyze the cleavage of C–N bond in amide compounds via hydrolytic or acyl transfer activity to generate the corresponding carboxylic acid (Wu et al., 2016). In recent decades, the application of amidases in bioremediation and biodegradation area is increasing (Wu et al., 2020). For example, the long and widespread use of the herbicide propanil (3, 4-dichloropropionanilide) was degraded by a novel amidase, PsaA from *Bosea* sp. P5 to generate the metabolite 3, 4-dichloroaniline (Zhang et al., 2023). Considering NEO degradation, *Microvirga flocculans* CGMCC 1.16731 was reported to degrade FLO to TFNG-AM and TFNG, mediated by a nitrile hydratase/amidase system. Two amidases, AmiA and AmiB, were shown to catalyze this reaction (Zhao et al., 2021a). An Asp-tRNAAsn/Glu-tRNAGln amidotransferase A subunit-like amidase, AmiD, was discovered to play the same role in Var*iovorax boronicumulans* CGMCC 4969, converting TFNG-AM to TFNG. Amidases can be classified into three categories, including amidase signature family, acet-amidase/formamidase family, and nitrilase superfamily (Wu et al., 2020). AmiA, AmiB and AmiD all contained the highly conserved catalytic triad variants Ser-Ser-Lys, belonged to the amidase signature family. The key amino acid residue Val154 in AmiD was identified by homology modeling and structural alignment and the mutant AmiD V154G showed a 3.08-fold increase in activity toward TFNG-AM compared with the wild-type AmiD. Additionally, AmiD is induced by the substrate TFNG-AM, and a member of the AraC family of regulators encoded upstream of the *amiD* gene was discovered. AraC is a transcriptional regulator of *araBAD*. qPCR analysis showed that the expression level of *amiD* in *V. boronicumulans* CGMCC 4969 cells cultured with the addition of 1 g/L arabinose was 1.93-fold than that without arabinose addition, indicating AraC plays an important role in regulating AmiD expression and therefore affects the activity of conversion of TFNG-AM to TFNG (Yu et al., 2024). The main degradation intermediate of FLO is TFNA-AM, which could be further degraded by *Pseudomonas stutzeri* CGMCC 22915 amidase, *Ps*AmiA, to TFNA. Although *Ps*AmiA also belongs to the AS family, it showed no activity toward TFNG-AM, which has a similar structure to TFNA-AM (Jiang H. Y. et al., 2023).

Amidases are often coupled with NHases, collectively constituting a nitrile hydratase/amidase system, which functions in the hydrolysis of nitriles to amide and carboxylic acid compounds. However, for ACE degradation, in spite of abundant NHases have been reported to be capable of degrading ACE to IM 1–2, but no matched amidases have been discovered that hydrolyze IM 1–2 to the corresponding carboxylic acid metabolite. The metabolite IM 1–4 has been reported to be the main intermediate during ACE metabolism in microorganisms, spinach and honeybees. The underlying molecular mechanism was investigated by Yang et al. (2020) who showed that a novel amidase (AceAB) in *Pigmentiphaga* sp. strain D-2, was responsible for the cleavage of the ACE C–N bond to generate IM 1–4. Unusually, AceAB is composed of two subunits, *α*-(AceA) and *β*-subunits (AceB), whereas amidases usually consist of a single subunit. Despite AceAB exhibiting high amino acid sequence identity to the α-and β-subunits of *Paracoccus aminophilus N*, *N*-dimethylformamidase, it showed no activity toward *N, N*-dimethylformamide or its structural analogs, which indicated its specificity for ACE.

### 3.4 Nitrilases

Nitrilases (EC 3.5.5.1) directly hydrolyze nitrile compounds into carboxylic acids and ammonia, and serves as the solitary branch of nitrile hydrolase/amidase superfamily (Zhou et al., 2024). Nitrilases have great value in production of important carboxylic acid compounds, such as nicotinic acid, iminodiacetic acid, acrylic acid (Chen et al., 2019). For the past few years, the application of nitrilases in biodegradation and bioremediation has also attracted much attention. Benzonitrile herbicides are widely used in agriculture to eliminate the weeds, which results in the environmental persistence. With the help of bioinformatic analysis, *Corynebacterium glutamicum* nitrilase-3 was identified; it can degrade benzonitrile herbicides such as dichlobenil, bromoxynil, and chloroxynil (Amrutha and Nampoothiri, 2022).

Regarding NEO degradation, Yang et al. (2019) reported the isolation of *Alcaligenes faecalis* CGMCC 17553, which can degrade FLO to TFNG and TFNG-AM. The genome of strain CGMCC 17553 contains five nitrilases, but only NitA and NitD have the ability to degrade FLO. Purified NitA catalyzed conversion of FLO into both TFNG and TFNG-AM, while NitD produced only TFNG-AM. Homology modeling analysis of the CGMCC 17553 NitA showed Glu-48, Lys-133, and Cys-167 constituted the catalytic triad and Glu-42, Lys-129, and Cys-163 made up the catalytic triad of NitD. In Var*iovorax boronicumulans* CGMCC 4969, nitrilases NitA and NitB both degraded FLO to TFNG and TFNG-AM (Jiang et al., 2022). Some research has focused on how to redesign such bifunctional nitrilases to enhance the hydration activity of nitrilase for amide formation, because compared with the traditionally used nitrile hydratases, nitrilases feature superior regioselectivity, stereoselectivity, and a broad substrate spectrum. Sosedov and Stolz (2014) mutated key residues W188 and N206 of NitEBC191 in *Pseudomonas fluorescens* EBC191, and the mutants W188L and N206K increased the amide ratio by up to 82 and 67%, respectively, but their relative activities decreased markedly. To improve the relative activity of amide formation, Sun et al. (2023) succeeded in generating a mutant of nitrilase NIT6803 from *Synechocystis* sp. PCC6803 (G101K/Q192H/I201M) that showed hydration activity of 98.5% and recovered the relative activity to 82.6% compared with the wild-type, which expands the toolbox for nitrilase-catalyzed amide formation. The FLO-degrading enzymes NitA and NitD from *Alcaligenes faecalis* CGMCC 17553 are a good choice of model enzymes for the study and development of nitrilase-catalyzed amide formation.

## 4 Toxicity of ACE and FLO and their degradation products

The extensive application of ACE in agriculture has adverse effects on non-target organisms (Zhao et al., 2024). Earthworms are an index of soil ecosystem health (Blouin et al., 2013). Siregar et al. (2024) investigated the behavior toxicities of *Eisenia fetida* exposed to different concentrations of ACE and observed decreased locomotion and altered movement orientation and complexity. ACE is highly water soluble, with residual concentrations of up to 0.41 mg/L in surface water, posing risks to aquatic organisms. Study of the acute toxicity of ACE to *Xenopus laevis* tadpoles revealed oxidative stress, bioconcentration and disruption of metabolism (Chen et al., 2024).

The degradation products of ACE are largely persistent in soil, water, honeybees, mice, and crops, and their impacts must be assessed. Iwasa et al. (2004) conducted laboratory bioassays to determine the contact toxicity of ACE and its metabolites toward honeybees. They found that ACE exhibited low toxicity (LD_50_ value of 7.1 mg/bee) and its degradation products IM 2–1, IM 0, and IC 0 produced no mortality at 50 mg/bee. The products IM 2–1 and IM 0 are catalyzed by the P450s, which indicates P450s are an important detoxification mechanism for ACE. Hano et al. (2017) investigated the toxicological effects of ACE degradation products IM 2–1 and IC 0 to *Marsupenaeus japonicas* (kuruma shrimp), and found those two metabolites were less toxic than the parent compound ACE. IC 0 is generated by the oxidation of ACE metabolites IM 0 or IM 1–4 and is a common intermediate metabolite of another NEO, IMI. Kai et al. (2023) assessed the developmental processes of medaka embryos exposed to neonicotinoid metabolite IC 0, and discovered that embryos exposed to 80 and 160 mg/L IC 0 showed no abnormalities until day 7 of exposure, but on day 8 of exposure, sudden embryo death was observed, which showed the potential long-term impact of the IC 0. The mixed ACE degradation products IM 1–2, IM1-4, and IC 0 formed by *Rhodococcus* sp. BCH2 were applied in toxicological analysis with respect to genotoxicity, antioxidant enzymes, lipid peroxidation, and protein oxidation using silkworm as the model animal, and those metabolites were less toxic than ACE (Phugare and Jadhav, 2015). The pharmacophore group cyanoimine (=N–CN) plays an important role in the insecticidal activity and toxicity of ACE and its decomposition resulted in the decreased toxicity. In addition, the selectivity of NEOs for insect nicotinic acetylcholine receptors (nAChRs) also be attributable to the pharmacophore groups nitroimine (=N–NO_2_) and cyanoimine (=N–CN), which have a much higher affinity for insects, in contrast to vertebrate. However, the loss of the nitro or cyano group to form the imine metabolite (=NH) can completely reverse the selective toxicity of NEOs (Tomizawa and Casida, 2003). For example, guanidine IMI has been reported to exhibit higher levels of toxicity toward mammals than IMI (Sahoo and Singh, 2014). The reported microbial degradation products of ACE are less toxic to invertebrates than ACE itself, providing options to lower the environmental risk of ACE residues.

Residues of FLO and its metabolites have been found in many harvested crops, and may enter the food chain (Zhang et al., 2019; Hengel and Miller, 2007). Thus, toxicity assessments of FLO and its metabolites are vital. Ghelichpour et al. (2019) evaluated the lethal toxicity and stress signs of FLO to *Cyprinus carpio* and some behavioral changes include hyperexcitement, erratic swimming, dark coloration, loss of equilibrium and lethargy were observed after suffered different intensity treatment. FLO also exhibits toxic effects toward mammals. The LD_50_ for rabbit is 180 mg/kg body weight (Muafia et al., 2022). Sabry et al. (2018) conducted the genetic risk evaluation of the FLO on mice’s genome as a monitor for detection the toxicity, mutagenicity and carcinogenic influence that human and livestock exposed. The results revealed that the high dose of FLO caused DNA degradation and severe genomic damage in treated mice. However, relevant evaluation of FLO metabolites is limited. TFNA-AM is a main degradation intermediate of FLO; it shows low mammalian toxicity, with an LD_50_ of >2000 mg/kg in mice (Taylor-Wells et al., 2018). Toxicity assessments of other common degradation products of FLO in plants and microorganisms, such as TFNA, TFNG-AM, TFNG are needed urgently.

## 5 Conclusion and prospects

This review summarizes the microbial degradation mechanisms of ACE and FLO, from the aspects of degradation pathways and the associated enzymes including cytochrome P450s, nitrile hydratases, amidases, and nitrilases. In addition, the toxicity assessments of ACE, FLO and their metabolites are reviewed. However, some challenges still need to be addressed. Firstly, for the increasing anxiety of co-contamination of NEOs, remolding microorganisms with elevated potential to degrade multiple NEOs using metabolic engineering methods are expectable. Besides, technologies such as genomics, transcriptomics, metagenomics, metabolomics and proteomics should be applied to gain information such as about the regulation of expression of correlated NEO degradation genes in microorganisms. To date, most research into microbial degradation of ACE and FLO has applied single bacterial strains, which do not achieve complete mineralization. Moreover, in the process of *in-situ* remediation, single degradation strains often suffer from metabolic burden and environmental pressure, resulting in decreased degradation rates. Consequently, the design and application of microbial consortia may accelerate detoxification and improve the survival of individual microbes via syntrophic and synergistic interactions.
